# Axl Inhibitor R428 Enhances TRAIL-Mediated Apoptosis Through Downregulation of c-FLIP and Survivin Expression in Renal Carcinoma

**DOI:** 10.3390/ijms20133253

**Published:** 2019-07-02

**Authors:** Seon Min Woo, Kyoung-jin Min, Seung Un Seo, Shin Kim, Peter Kubatka, Jong-Wook Park, Taeg Kyu Kwon

**Affiliations:** 1 Department of Immunology, School of Medicine, Keimyung University, Daegu 42601, Korea; 2 Department of Medical Biology, Jessenius Faculty of Medicine, Comenius University in Bratislava, 03601 Martin, Slovakia; 3 Department of Experimental Carcinogenesis, Division of Oncology, Biomedical Center Martin, Jessenius Faculty of Medicine, Comenius University in Bratislava, 03601 Martin, Slovakia

**Keywords:** Axl, TRAIL, c-FLIP, survivin, apoptosis

## Abstract

R428, a selective small molecule Axl inhibitor, is known to have anti-cancer effects, such as inhibition of invasion and proliferation and induction of cell death in cancer cells. The Axl receptor tyrosine kinase is highly expressed in cancer cells and the level of Axl expression is associated with survival, metastasis, and drug resistance of many cancer cells. However, the effect of Axl inhibition on overcoming anti-cancer drugs resistance is unclear. Therefore, we investigated the capability of Axl inhibition as a therapeutic agent for the induction of TRAIL (tumor necrosis factor-related apoptosis-inducing ligand) sensitivity. In this study, R428 markedly sensitized cancer cells to TRAIL-induced apoptotic cell death, but not in normal human skin fibroblast (HSF) and human umbilical vein cells (EA.hy926). Moreover, knockdown of Axl by siRNA also increased TRAIL-induced apoptosis. R428 decreased c-FLIP proteins levels via induction of miR-708 expression and survivin protein levels at the post-translational level, and we found that knockdown of Axl also decreased both c-FLIP and survivin protein expression. Overexpression of c-FLIP and survivin markedly inhibited R428 plus TRAIL-induced apoptosis. Furthermore, R428 sensitized cancer cells to multiple anti-cancer drugs-mediated cell death. Our results provide that inhibition of Axl could improve sensitivity to TRAIL through downregulation of c-FLIP and survivin expression in renal carcinoma cells. Taken together, Axl may be a tempting target to overcome TRAIL resistance.

## 1. Introduction

Tumor necrosis factor-related apoptosis-inducing ligand (TRAIL) specifically induces apoptosis of most tumor cells, but not of normal cells [1,2]. However, many cancer cells reveal the resistance of TRAIL through increased decoy receptors and decreased death receptor (DR), anti-apoptotic Bcl-2 and IAP family proteins, and c-FLIP [3,4,5]. Due to the defects of cancer therapy using TRAIL, many studies suggest a way to overcome TRAIL resistance through modulation of protein expression, which are related with anti-cancer drugs resistance. Combined treatment with chemotherapeutic agent is one of the ways to increase the sensitivity to anti-cancer drugs [6]. Therefore, identification of TRAIL sensitizers is needed to propose a way to overcome TRAIL resistance.

The Axl receptor tyrosine kinase is one of the TAM family members (Tyro3, AXL, and Mer) and growth arrest specific 6 (Gas 6) is a ligand for Axl. Binding of ligand resulted in dimerization of extracellular domains and auto-phosphorylation of Axl at Tyr702/703 residues [7,8,9]. Activation of Gas6/Axl signaling pathway plays important roles in cell angiogenesis, epithelial-to-mesenchymal transition (EMT), invasion and survival through activation of multiple cellular signaling pathway, including PI3K/Akt, MAPK/ERK, and STAT3 signaling [10,11,12,13,14]. Since Axl is overexpressed in many cancer cells compared to normal cells [15,16,17], it resulted in the poor prognosis of cancer patients [16,18,19,20]. A selective small molecule Axl inhibitor, R428, currently entered phase II clinical trials for multiple cancers [21]. Previous reports examined that R428 inhibits cancer cell proliferation, migration and metastasis [22,23,24,25]. Moreover, R428 overcomes resistance to chemotherapeutic agents of many cancer cells. For example, R428 reverses sensitivity to paclitaxel of uterine serious cancer cells, metformin of prostate cancer, and erlotinib of head and neck cells [26,27]. R428 also overcomes chemoresistance through Akt/GSK-3β/β-catenin-mediated ZEB1 inhibition against doxorubicin-resistant MCF-7 breast cancer cells [28]. In addition, R428 increases gemcitabine sensitivity via tumor immune suppression in pancreatic cancer [29]. Even though several reports show the effect of R428 on overcoming drug resistance, the molecular mechanisms of R428 have not been elucidated.

In this study, we show that inhibition of Axl overcomes TRAIL resistance through downregulation of c-FLIP and survivin expression in cancer cells.

## 2. Results

### 2.1. Inactivation of Axl Sensitizes Cancer Cells to TRAIL-Induced Apoptosis, but Not Normal Cells

**Figure 1:**
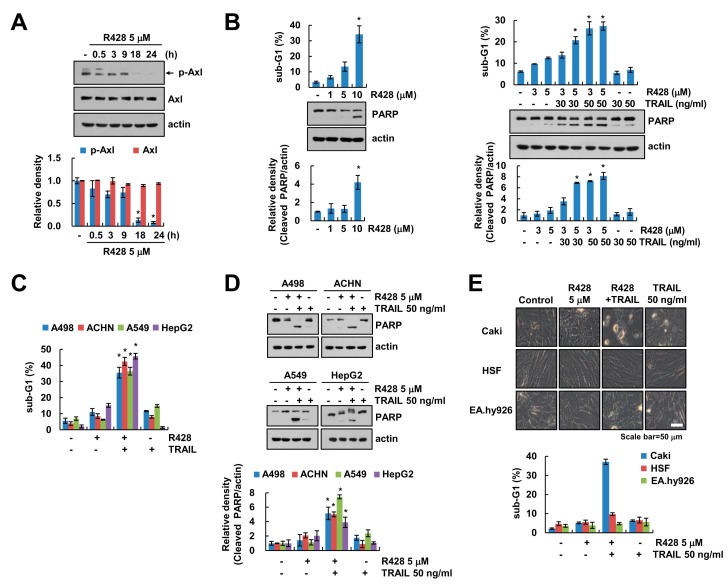
Inhibition of Axl increases TRAIL (tumor necrosis factor-related apoptosis-inducing ligand)-mediated apoptosis of cancer cells, but not normal cells. (**A**) Caki cells were treated with Axl inhibitor (R428) for the indicated time kinetics. (**B**) Caki cells were treated with 1–10 µM R428 for 24 h (left panel). Caki cells were treated with R428 (3, 5 µM) in the presence or absence of TRAIL (30, 50 ng/mL) for 24 h (right panel). (**C**,**D**) Indicated cancer cells were treated with 5 µM R428 alone, 50 ng/mL TRAIL alone or R428 plus TRAIL for 24 h. (**E**) Caki, HSF and EA.hy926 cells were treated with 5 µM R428, 50 ng/mL TRAIL or R428 plus TRAIL for 24 h. The cell morphology was examined using interference light microscopy. The sub-G1 population and protein expression were detected by flow cytometry (**B**,**C**,**E**) and Western blotting (**A**,**B**,**D**), respectively. The band intensity was quantified using Image J (**A**,**B**,**D**). The values in graph (**A**–**E**) represent the mean ± SEM of three independent experiments. * *p* < 0.05 compared to the control. Black arrow (**A**) indicated specific band of p-Axl.

To examine the effect of Axl specific inhibitor (R428) on activation of Axl, we checked phosphorylation of Axl at Tyr702 through immunoblotting. We found that R428 markedly decreased phospho (p)-Axl expression (Figure 1A). Recently, Axl inhibition enhances the antitumor effect of docetaxel both in vitro and in vivo [30]. We investigated the molecular mechanisms of the sensitizing effect of R428 on TRAIL-induced cell death in renal cancer cells. R428 dose-dependently induced sub-G1 population and Poly (ADP-ribose) polymerase (PARP) cleavage (Figure 1B, left panel). We chose 5 µM concentration of R428 for further studies because its dose did not induce apoptosis much. Combinations treatment with R428 and TRAIL markedly induced apoptosis, but R428 alone and TRAIL alone did not increase apoptosis (Figure 1B, right panel). Moreover, R428 enhanced TRAIL-induced apoptosis in various cancer cells (human renal cancer A498 and ACHN, human lung cancer A549, and human hepatocellular carcinoma HepG2 cells) (Figure 1C,D). However, combined treatment with R428 and TRAIL did not increase morphological apoptotic bodies and apoptotic cell death in normal human skin fibroblast (HSF) and normal human umbilical vein cells (EA.hy926) (Figure 1E). Therefore, these results suggest that inhibition of Axl enhances TRAIL-induced apoptosis in cancer cells, but not in normal cells.

### 2.2. R428 Increases TRAIL-Mediated Apoptosis in Caspase-Dependent Manner Via Downregulation of c-FLIP and Survivin Expression

**Figure 2:**
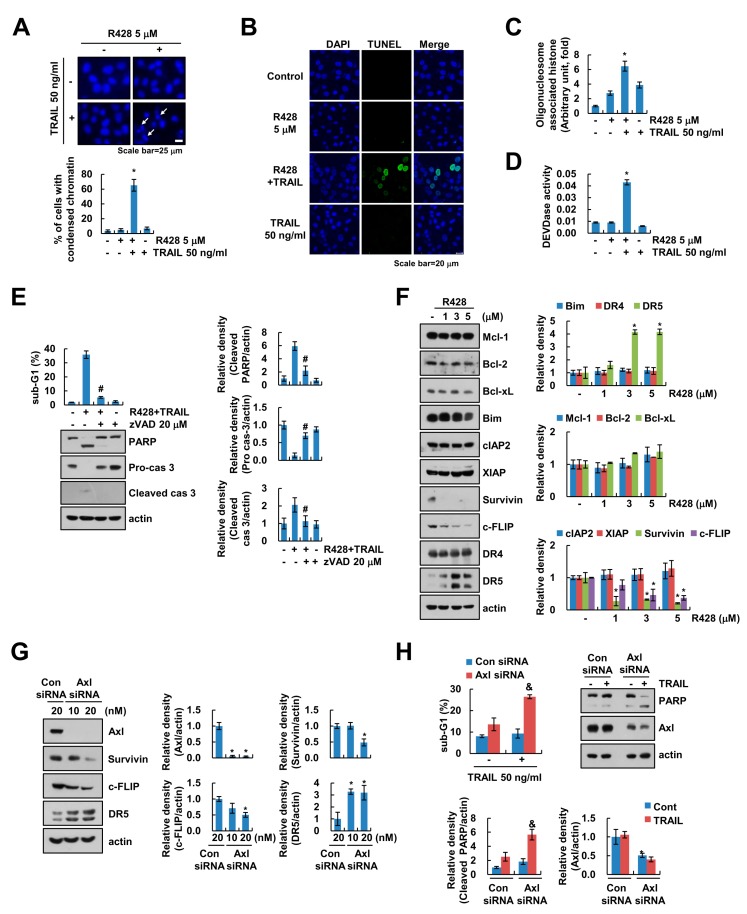
R428 increases caspase-dependent apoptosis through upregulation of DR5 expression and downregulation of c-FLIP and survivin expression. (**A**–**D**) Caki cells were treated with 5 µM R428 alone, 50 ng/mL TRAIL alone or R428 plus TRAIL for 24 h. DAPI staining (**A**), TUNEL staining (**B**), cytoplasmic histone-associated DNA fragments (**C**), and DEVDase (caspase-3) activity (**D**) were examined. Condensed chromatin rate determined by counting the number of apoptotic cells (**A**). (**E**) Caki cells were treated with 5 µM R428 plus 50 ng/mL TRAIL in the presence or absence of 20 µM z-VAD for 24 h. (**F**) Caki cells were treated with various concentrations of R428 for 24 h. (**G**,**H**) Caki cells were transfected with control (Con) or Axl siRNA for 24 h, and then cells were further incubated for 24 h (**G**) or were treated with 50 ng/mL TRAIL for 24 h (**H**). The sub-G1 population and protein expression were detected by flow cytometry (**E**,**H**) and Western blotting (**E**–**H**), respectively. The band intensity was quantified using Image J (**E**–**H**). The values in graph (**A**,**C**–**H**) represent the mean ± SEM of three independent experiments. * *p* < 0.01 compared to the control. # *p* < 0.01 compared to the R428 plus TRAIL. & *p* < 0.05 compared to the TRAIL in control siRNA.

We found that combined treatment with R428 and TRAIL induced the nuclear chromatin condensation and DNA fragmentation (Figure 2A,C). Moreover, R428 plus TRAIL increased TUNEL-positive cells (Figure 2B). To prove the involvement of caspase activation in R428 plus TRAIL-induced cell death, we checked caspase-3 activity and used pan-caspase inhibitor, z-VAD-fmk (z-VAD). As shown in Figure 2D, combined treatment with R428 and TRAIL increased caspase-3 activation. Furthermore, z-VAD markedly inhibited combined treatment-induced apoptosis, and inhibited cleavage of caspase-3 (Figure 2E). Next, we investigated which apoptosis-related proteins are regulated by R428 treatment. R428 induced upregulation of DR5 and downregulation of c-FLIP and survivin expression, whereas expression of other apoptosis related proteins (Mcl-1, Bcl-2, Bcl-xL, Bim, cIAP2, XIAP, and DR4) was not changed (Figure 2F). As shown in Figure 2G, knockdown of Axl by siRNA also induced up-regulation of DR5 and downregulation of c-FLIP and survivin (Figure 2G). Furthermore, knockdown of Axl sensitized Caki cells to TRAIL-mediated apoptosis (Figure 2H). These data indicate that inhibition of Axl enhances caspase-dependent TRAIL-induced apoptosis through modulation of apoptosis-related proteins expression.

### 2.3. Downregulation of c-FLIP Is Associated with Induction of Apoptosis by Combined Treatment with R428 and TRAIL

**Figure 3:**
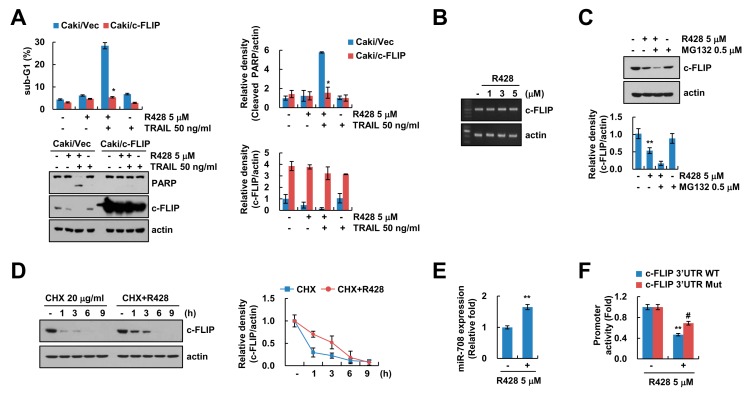
Downregulation of c-FLIP is associated with induction of TRAIL-mediated apoptosis by R428. (**A**) Vector cells and c-FLIP-overexpressing cells (Caki/c-FLIP) were treated with 5 µM R428, 50 ng/mL TRAIL or R428 plus TRAIL 24 h. (**B**) Caki cells were treated with various concentrations of R428 for 24 h. The levels of mRNA were examined using RT-PCR. (**C**) Caki cells were treated with 5 µM in the presence or absence of 0.5 µM MG132 for 24 h. (**D**) Caki cells were treated with 5 µM R428 in the presence or absence of 20 µg/ml cycloheximide (CHX) for the indicated time kinetics. (**E**) Caki cells were treated with 5 µM R428 for 24 h, and then miR-708 expression was examined by qPCR. (**F**) For luciferase reporter assay, Caki cells transfected with c-FLIP 3′UTR wild-type (WT) and mutant (Mut), and cells were treated with 5 µM R428 for 24 h. The sub-G1 population and protein expression were detected by flow cytometry (**A**) and Western blotting (**A**,**C**,**D**), respectively. The band intensity was quantified using Image J (**A**,**C**,**D**). The values in graph (**A**,**C**–**F**) represent the mean ± SEM of three independent samples. * *p* < 0.01 compared to R428 plus TRAIL-treated Caki/Vec. ** *p* < 0.01 compared to control. # *p* < 0.05 compared to the R428-treated in c-FLIP 3′UTR WT.

Next, we investigated whether downregulation of c-FLIP is critical for R428-mediated enhancement of TRAIL sensitivity. Using c-FLIP overexpressed stable cells, overexpression of c-FLIP significantly inhibited apoptosis and PARP cleavage by R428 plus TRAIL treatment (Figure 3A). Previous studies reported that expression of c-FLIP is modulated at the transcriptional levels or ubiquitin-proteasome-mediated post-translational levels [31]. Therefore, we first examined the effect of R428 on c-FLIP mRNA expression. R428 did not change c-FLIP mRNA expression (Figure 3B). Next, to identify the relation of ubiquitin-proteasome-mediated post-translational modification, we used MG132, a proteasome inhibitor. However, MG132 did not reverse c-FLIP downregulation by R428 (Figure 3C). Moreover, when we checked c-FLIP protein stability through use of a protein biosynthesis inhibitor, cycloheximide (CHX), R428 plus CHX did not induce more degradation of c-FLIP expression compared to CHX alone (Figure 3D). These results indicate that ubiquitin-proteasome pathways are not involved in downregulation of c-FLIP expression in R428-treated cells.

Ours and other groups previously reported that various microRNAs (miRNAs) including miR-126, miR-708, and miR-512-3p regulate levels of c-FLIP expression [32,33,34]. The miR-708 is known to target c-FLIP expression by binding to the nucleotides 2489 to 2495 in 3′-untranslated region (3′UTR) of c-FLIP [33,35]. As shown in Figure 3E, R428 increased miR-708 levels in Caki cells (Figure 3E). To assess the role of miR-708 on R428-mediated inhibition of c-FLIP expression, we produced mutant construct of c-FLIP 3′UTR that interfere miR-708 binding [35]. The c-FLIP 3′UTR mutant reversed inhibition of c-FLIP 3′UTR luciferase activity by R428 treatment (Figure 3F). Therefore, these results suggest that downregulation of c-FLIP by R428 plays a critical for increase of TRAIL-sensitivity, and R428 inhibits c-FLIP expression via the increase of miR-708 expression, and it can be involved in TRAIL sensitization.

### 2.4. Inhibition of Survivin by R428 Contributes to TRAIL Sensitization

**Figure 4:**
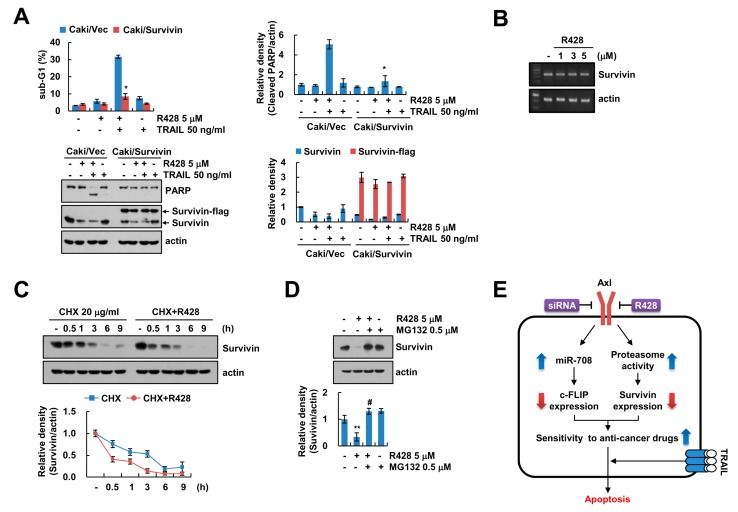
Downregulation of survivin by R428 contributes to induction of TRAIL-mediated apoptosis. (**A**) Vector cells and survivin-overexpressing cells (Caki/Survivin) were treated with 5 µM R428, 50 ng/mL TRAIL or R428 plus TRAIL for 24 h. (**B**) Caki cells were treated with various concentrations of R428 for 24 h. The levels of mRNA were examined using RT-PCR. (**C**) Caki cells were treated with 5 µM R428 in the presence or absence of 20 µg/mL CHX for the indicated time kinetics. (**D**) Caki cells were treated with 5 µM in the presence or absence of 0.5 µM MG132 for 24 h. (**E**) The diagram showing the mechanism of R428-mediated TRAIL sensitization. The sub-G1 population and protein expression were detected by flow cytometry (**A**) and Western blotting (**A**,**C**,**D**), respectively. The band intensity was quantified using Image J (**A**,**C**,**D**). The values in graph (**A**,**C**,**D**) represent the mean ± SEM of three independent samples. * *p* < 0.01 compared to R428 plus TRAIL-treated Caki/Vec. ** *p* < 0.01 compared to the control. # *p* < 0.01 compared to the R428.

Since R428 inhibited survivin protein levels (Figure 2F), we investigated the role of survivin in sensitization of Caki cells to R428 plus TRAIL-induced apoptosis using survivin overexpressed Caki cells. Ectopic expression of survivin markedly prevented R428 plus TRAIL-induced apoptosis (Figure 4A). To elucidate the molecular mechanisms underlying R428-mediated survivin downregulation, we checked the potentiality for transcriptional regulation. However, survivin mRNA was not changed by R428 treatment (Figure 4B). Therefore, we examined the regulation of survivin by R428 at post-translational levels. We found that R428 reduced the stability of survivin (Figure 4C), and proteasome activity was involved in downregulation of survivin expression in R428-treated cells (Figure 4D).

### 2.5. The Effect of R428 on Anti-Cancer Drugs-Mediated Apoptosis

Previous studies demonstrate that expression and activation of Axl contributes to the drug resistance of cancer cells [30,36]. To investigate that the inactivation of Axl could sensitize cancer cells to anti-cancer drugs-mediated apoptosis, Caki cells were treated with anti-cancer drugs in the absence or presence of R428. R428 increased sensitivity to anti-cancer drugs, followed by the induction of apoptosis (Figure S1). Thus, these data indicate that inhibition of Axl sensitizes cancer cells to anti-cancer drugs-mediated apoptosis including TRAIL.

## 3. Discussion

Our data indicated that inhibition or knockdown of Axl sensitized the human renal carcinoma Caki cells to TRAIL-mediated apoptosis through downregulation of c-FLIP and survivin expression. Inhibition of Axl by R428 induced downregulation of c-FLIP via induction of miR-708, and proteasome-dependent downregulation of survivin (Figure 4E). In addition to TRAIL, we found that R428 enhanced multiple anti-cancer drugs-mediated apoptosis in cancer cells (Figure S1). Therefore, inactivation of Axl could be as therapeutic target capable of increasing TRAIL sensitivity.

Since increased DR expression is important for TRAIL sensitization [4], we examined protein levels and cellular surface expression of DR5. Even though R428 increased DR5 protein expression (Figure 2E), DR5 expression on the surface was not increased in R428-treated cells (Woo et al., unpublished). Thus, the increased expression of DR5 was excluded from the protein that regulates sensitivity to TRAIL by R428.

In this study, we first identified that inhibition or knockdown of Axl induced downregulation of c-FLIP expression (Figure 2F,G). Downregulation of c-FLIP by R428 was independent of mRNA expression (Figure 3B). Therefore, we expected ubiquitin-proteasome pathway to be involved in R428-mediated downregulation of c-FLIP expression. However, proteasome inhibitor (MG132) did not restore R428-mediated c-FLIP downregulation, and R428 did not alter protein stability (Figure 3C,D). These data indicate that downregulation of c-FLIP expression by R428 was not modulated at the transcriptional and post-translational levels. The miRNA interrupts activation of gene expression by binding 5′UTR or 3′UTR regions, and previous studies reported that increased miR-126, miR-708, and miR-512-3p repress c-FLIP expression [32,33,34]. Moreover, we also previously reported the inhibition of c-FLIP expression by miR-708 [35]. As shown in Figure 3E, R428 induced upregulation of miR-708 expression (Figure 3E), and the luciferase activity of the reporter vector containing a mutated miR-708 binding sites 3′UTR in c-FLIP was less affected by R428 (Figure 3F). These finding suggest that miR-708 contributed to inhibition of c-FLIP expression by R428. However, further studies will need to approach whether other miRNA, miR-126, and miR-512-3p are associated with R428-mediated downregulation of c-FLIP expression.

Downregulation of survivin is regulated by ubiquitin-proteasome system through activation of E3 ligases, such as XIAP [37]. While R428 inhibited survivin protein expression, XIAP expression was not changed (Figure 2E). Moreover, ubiquitin-mediated protein regulation is negatively modulated by deubiquitinating enzymes (DUBs), and DUBs are classified to five groups, including ubiquitin-specific proteases (USPs), ovarian tumor proteases (OTUs), ubiquitin C-terminal hydrolases (UCHs), Machado–Joseph disease proteases, and JAB1/MPN/Mov34 metalloenzymes (JAMMs) [38,39]. Previously, since we reported that downregulation of STAMBPL1 is critical to survivin degradation [40], we examined the effect of STAMBPL1 on R428-mediated downregulation of survivin. Overexpression of STAMBPL1 did not reverse downregulation of survivin expression by R428 (Woo et al., unpublished). Therefore, further studies are required to explore the molecular mechanism for R428-induced survivin downregulation.

Recently, Chen et al. reported that R428 accumulates autophagosome and lysosome via inhibition of lysosomal acidification, resulted in induction of apoptosis in human non-small cell lung carcinoma cell lines [41]. Therefore, we also examined whether R428 modulates autophagy in renal carcinoma Caki cells. When we treated R428 in human renal carcinoma Caki cells, cytoplasmic vacuolization and LC3 II conversion were increased (Figure S2A,B). To examine the regulation of autophagic flux by R428, we used autophagy inhibitors (3-Methyladenine, chloroquine, and bafilomycin A1). 3-MA decreased R428-mediated LC3 II conversion, and chloroquine and bafilomycin A1 increased R428-mediated LC3 II conversion (Figure S2C). These results indicated that R428 increased autophagic flux in renal cancer cells. R428 sensitized cancer cells to TRAIL-induced apoptosis through downregulation of c-FLIP and survivin. However, autophagy inhibitors did not reverse R428-mediated downregulation of c-FLIP and survivin expression (Figure S2D). Therefore, induction of sensitization cancer cells to TRAIL by R428 may not be related with induction of autophagy. Further research will be needed on what function the autophagy induction by R428 has in cancer cells.

Collectively, our findings are of potential clinical relevance and provide the molecular mechanism in the development of combination therapy using Axl inhibitor to sensitize cancer cells to TRAIL-induced apoptosis. Inhibition of the expression and activity of Axl may be an effective target for increasing TRAIL sensitivity and it could eventually contribute to the cancer treatment.

## 4. Materials and Methods

### 4.1. Cell Culture and Transfection

Caki, ACHN, A498, A549, and HepG2 cells were obtained from American Type Culture Collection (Manassas, VA, USA). HSF cells were purchased from Korea Cell Line Bank (Seoul, Korea), and EA.hy926 cells were a gift from T.J. Lee (Yeungnam University, Daegu, Korea). Cells were grown in appropriate medium supplemented with 10% FBS (Welgene, Gyeongsan, Korea), 1% penicillin-streptomycin and 100 µg/mL gentamycin (Thermo Fisher Scientific, Waltham, MA, USA). For constructing stable cell lines, Caki cells were transfected using Lipofectamine™ 2000 (Invitrogen, Carlsbad, CA, USA) with the pcDNA3.1(+)/c-FLIP, pcDNA3.1(+)/survivin-flag or pcDNA3.1(+) plasmids. These plasmids were transduced for 24 h and cells were selected by 700 µg/mL G418 (Invitrogen). For knockdown of gene by siRNA, Lipofectamine® RNAiMAX Reagent (Invitrogen) was used in Caki cells. Immunoblot analysis was performed to examine protein expression.

### 4.2. Reagents, Antibodies, siRNAs and Plasmids

Selleckchem supplied R428 and cisplatin (Houston, TX, USA), and R&D system supplied recombinant human recombinant TRAIL and z-VAD-fmk (Minneapolis, MN, USA). Sigma Chemical Co. provided MG132, cycloheximide, carboplatin, oxaliplatin, doxorubicin, and 5-FU (St. Louis, MO, USA). The primary antibodies were as follows: Anti-phospho-Axl (Y702) (Cell Signaling Technology, Beverly, MA, USA), anti-pro-caspase-3 (Cell Signaling Technology), anti-cleaved caspase-3 (Cell Signaling Technology), anti-PARP (Cell Signaling Technology), anti-Bcl-xL (Cell Signaling Technology), anti-DR5 (Cell Signaling Technology), anti-actin (Sigma Chemical Co.), anti-Axl (Santa Cruz Biotechnology, St. Louis, MO, USA), anti-Mcl-1 (Santa Cruz Biotechnology), anti-Bcl-2 (Santa Cruz Biotechnology), anti-cIAP2 (Santa Cruz Biotechnology), anti-Bim (BD Biosciences, San Jose, CA, USA), anti-XIAP (BD Biosciences), anti-survivin (R&D system, Minneapolis, MN, USA), anti-DR4 (Abcam, Cambridge, MA, USA), and anti-c-FLIP (Enzo Life Sciences, San Diego, CA, USA). The siRNAs were as follows: GFP (control) siRNA (Bioneer, Daejeon, Korea), Axl siRNA (Santa Cruz Biotechnology), and DR5 siRNA (Invitrogen).

### 4.3. Western Blotting

Cells were lysed in RIPA lysis buffer (20 mM HEPES and 0.5% Triton X-100, pH 7.6) and separated by 10% SDS-PAGE [42]. Proteins were transferred to nitrocellulose membranes (GE Healthcare Life Science, Pittsburgh, PO, USA) and checked using an Immobilon Western Chemiluminescent HRP Substrate (EMD Millipore, Darmstadt, Germany) for analysis protein expression.

### 4.4. FACS Analysis

For apoptosis analysis, cells were harvested and suspended in 100 µL of phosphate-buffered saline, and added to 200 µL of 95% ethanol. And then, cells were incubated in 1.12% sodium citrate buffer containing RNase at 37 °C for 30 min, added to 50 µg/mL propidium iodide, and analyzed using BD Accuri™ C6 flow cytometer (BD Biosciences).

### 4.5. DAPI (4′6-Diamidino-2-Phenylindole) for Nucleic Acid Staining

The cells were fixed with 1% paraformaldehyde. And then cells were washed with PBS and stained with 300 nM 4′,6′-diamidino-2-phenylindole solution (Roche, Basel, Switzerland) for 10 min. The cell morphology and condensation of the nucleus were examined by fluorescence microscope (Carl Zeiss, Jena, Germany).

### 4.6. TUNEL Staining

To detect the apoptosis, we used TUNEL assay. It was performed using the ApopTag Fluorescein In Situ Apoptosis Detection Kit (EMD Millipore, Darmstadt, Germany) according to the manufacturer’s recommendations.

### 4.7. DNA Fragmentation and DEVDase Activity Assay for Detection of Apoptosis

Caki cells were treated with R428 alone, TRAIL alone or R428 plus TRAIL. For DEVDase activity assay, cells were harvested and incubated with reaction buffer containing acetyl-Asp-Glu-Val-Asp p-nitroanilide (Ac-DEVD-pNA) substrate, as previously described [43]. To measure DNA fragmentation, we used cell death detection ELISA plus kit (Boehringer Mannheim, Indianapolis, IN, USA) according to the manufacturer’s recommendations. The reaction products were analyzed by spectrophotometry (BMG Labtech, Ortenberg, Germany) at 405 and 490 nm (reference wavelength).

### 4.8. Reverse Transcription Polymerase Chain Reaction (RT-PCR)

Total RNA was isolated with TriZol reagent (Life Technologies, Gaithersburg, MD, USA), and prepared cDNA using M-MLV reverse transcriptase (Gibco-BRL, Gaithersburg, MD, USA). For PCR, we used Blend Taq DNA polymerase (Toyobo, Osaka, Japan) with primers targeting c-FLIP, survivin and actin. The used primers were referred to previous studies [44].

### 4.9. Statistical Analysis

The data were analyzed using a one-way ANOVA and post-hoc comparisons (Student-Newman-Keuls) using the SPSS software (SPSS Inc., Chicago, IL, USA).

## 5. Conclusions

Our data suggested that inhibition of Axl sensitized cancer cells to TRAIL-mediated apoptosis via miR-708-mediated downregulation of c-FLIP expression and proteasome-dependent downregulation of survivin expression.

## Supplementary Materials

Supplementary materials can be found at https://www.mdpi.com/1422-0067/20/13/3253/s1.
