# Supplementary figures and images for: Axl Inhibitor R428 Enhances TRAIL-Mediated Apoptosis Through Downregulation of c-FLIP and Survivin Expression in Renal Carcinoma
Source: Int J Mol Sci. 2019 Jul 2;20(13):3253. doi: 10.3390/ijms20133253 (PMC6651098; doi:10.3390/ijms20133253)

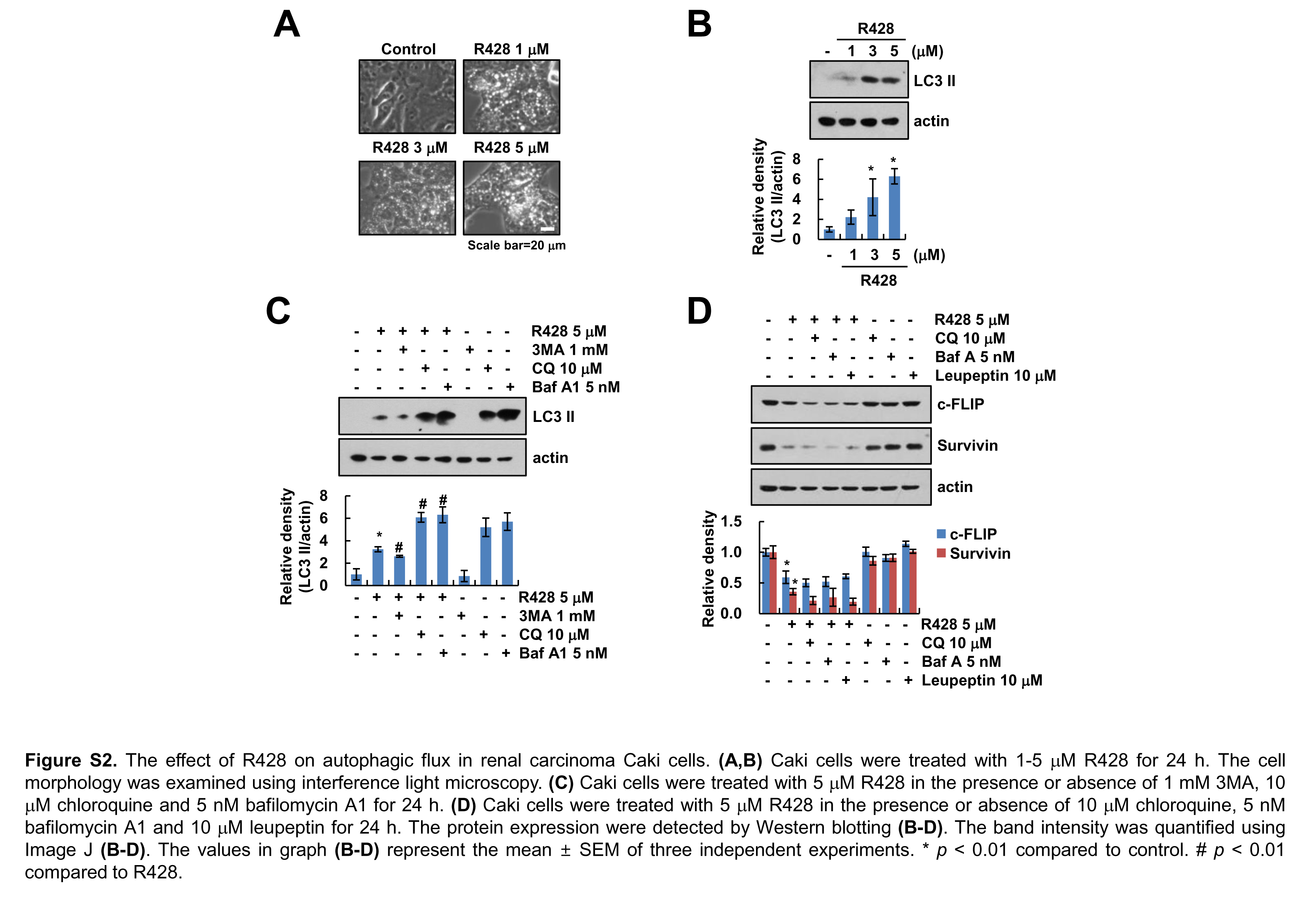

Supplement: Supplementary file 1 [file ijms-20-03253-s001.zip › Figure S2.tif]

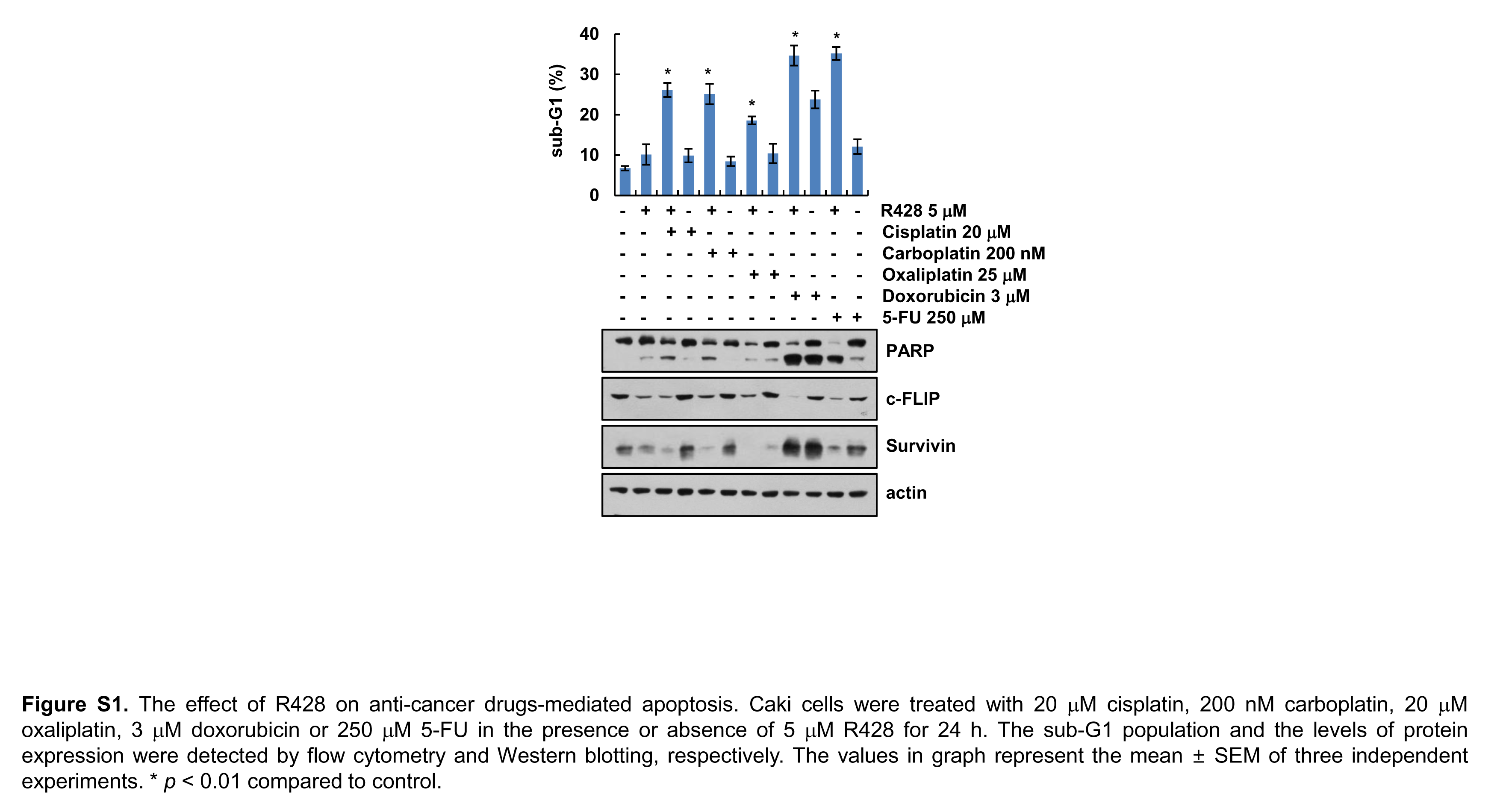

Supplement: Supplementary file 1 [file ijms-20-03253-s001.zip › Figure S1.tif]
